# Comparative analysis of alpha-fetoprotein, carbohydrate antigen 19-9, carcinoembryonic antigen, and prostate-specific antigen among conventional cigarette smokers, heated tobacco product users and quitters

**DOI:** 10.18332/tid/200890

**Published:** 2025-03-12

**Authors:** Dae-Hyun Kim, Seung-Wan Hong, Naeun Park

**Affiliations:** 1 Department of Family Medicine, Keimyung University School of Medicine, Daegu, Republic of Korea; 2 Chosun University School of Medicine, Daegu, Republic of Korea

**Keywords:** combustible cigarettes, heated tobacco products, quitters, tumor markers

## Abstract

**INTRODUCTION:**

The association of Heated Tobacco Products (HTPs) use on cancer-related biomarkers remains unclear. This study aimed to compare the levels of tumor markers, specifically alpha-fetoprotein (AFP), carbohydrate antigen 19-9 (CA 19-9), carcinoembryonic antigen (CEA), and prostate-specific antigen (PSA), between combustible cigarette (CC) smokers, any HTP users, and quitters.

**METHODS:**

This cross-sectional study compared tumor marker levels (AFP, CA 19-9, CEA, PSA) among 750 adult males: 250 CC smokers, 250 any HTP users, and 250 quitters. Data were collected from health screenings (2021–2022). Participants were aged >18 years with at least one year of smoking history.

**RESULTS:**

CEA was significantly higher in CC smokers (median: 2.4) than any HTP users (median: 2.0) and quitters (median: 1.6), with any HTP users exceeding quitters. PSA was higher in any HTP users (median: 0.86) than quitters (median: 0.74). No significant differences were observed in AFP and CA 19-9.

**CONCLUSIONS:**

HTP users exhibit lower CEA levels compared to conventional cigarette smokers, yet their levels remain higher than those of quitters. Additionally, quitters were found to have lower PSA levels than HTP users. Further research is needed to determine the reasons for these differences.

## INTRODUCTION

The adverse health effects of smoking combustible cigarettes (CCs) are well-established, with smoking being one of the primary causes of preventable diseases and premature deaths worldwide^1^. Tobacco smoke contains over 7000 chemicals, many of which are toxic and carcinogenic, significantly increasing the risk of cancers, particularly lung, liver, and colorectal cancers^2-4^. Consequently, smoking cessation has long been emphasized as a vital public health intervention to reduce these risks^5^.

In response to the dangers posed by CC smoking, heated tobacco products (HTPs) have been introduced as an alternative. HTPs heat tobacco at lower temperatures compared to CCs, thereby reducing combustion and the release of harmful chemicals associated with traditional smoking. This reduced combustion has been linked to lower emissions of harmful substances such as polycyclic aromatic hydrocarbons (PAHs) and carbon monoxide^6,7^. This feature has led to the widespread adoption of HTPs, particularly among smokers seeking a ‘safer’ alternative without fully quitting^8^.

However, the claim that HTPs are significantly less harmful than CCs remains a subject for debate. While HTPs may reduce some toxic exposures compared to conventional cigarettes, they are not risk-free^7^. Studies have demonstrated that HTPs still contain harmful chemicals, including carcinogens such as aldehydes and nitrosamines, although at lower levels than CCs^8-11^. Biomarkers of cancer risk, such as alpha-fetoprotein (AFP), carbohydrate antigen 19–9 (CA 19-9), carcinoembryonic antigen (CEA), and prostate-specific antigen (PSA), are crucial in evaluating the potential health impacts of these products.

AFP is primarily associated with hepatocellular carcinoma and testicular cancer, while CA 19–9 is linked to gastrointestinal cancers such as pancreatic cancer^12,13^. Elevated CEA levels indicate various malignancies, including lung and gastrointestinal cancers^13,14^. PSA is specifically used to assess prostate health and elevated levels may indicate prostate cancer^15^. Understanding how these biomarkers change in response to different forms of smoking or cessation is critical for evaluating the health risks and potential benefits of quitting.

This study aims to fill this gap by comparing the levels of key tumor markers (AFP, CA 19-9, CEA, and PSA) between a sample of combustible cigarette smokers, any HTP users, and quitters.

## METHODS

### Study design and population

This cross-sectional study included a total of 750 adult male participants, divided into three groups: 250 current combustible cigarette (CC) smokers, 250 any heated tobacco product (HTP) users, and 250 quitters. Participants were recruited during health screenings conducted between 2021 and 2022. Inclusion criteria required participants to be aged >18 years and to have smoked for at least one year before the study began. In this study, individuals with serious chronic conditions (such as cancer, cirrhosis, chronic obstructive pulmonary disease, cardiovascular disease, or cerebrovascular disease) or those with irregular smoking habits, were excluded. Any HTP users were defined as individuals who used HTPs exclusively or concurrently with CCs, regardless of the extent of concurrent CC smoking. Quitters are defined as subjects who have not smoked in the past 6 months to 10 years.

### Measures

The tumor markers analyzed were alpha-fetoprotein (AFP), carbohydrate antigen 19-9 (CA 19-9), carcinoembryonic antigen (CEA), and prostate-specific antigen (PSA). Blood collection was performed by two people in rotation, and each blood test was conducted using the electrochemiluminescence immunoassay (ECLIA) method. Demographic information, including age, BMI, smoking status, duration of smoking cessation (for quitters), duration of HTP use, smoking duration, was collected through self-reported questionnaires completed during the health screening visits. Due to incomplete responses for certain questions, the number of participants included in the analysis for each tumor marker varied, as not all participants had complete tumor marker measurements. This study was conducted after receiving approval from the Research Ethics Review Committee of Keimyung University Dongsan Hospital (2022-06-047).

The sample size for the study was determined using G*Power. Assuming a one-way ANOVA with three groups and an effect size of 0.25, the required sample size was calculated to be 252 subjects.

### Statistical analysis

Statistical analyses were performed using SPSS (version 30.0.0.0; IBM Corp., Armonk, NY, USA). The data analysis was conducted in multiple stages. Initially, descriptive statistics were computed to summarize participant characteristics, including age, BMI, smoking duration, and tumor marker levels for each group. Normality was assessed, and variables that were normally distributed were presented as mean and standard deviation, while non-normally distributed variables were presented as median and interquartile range (IQR). To compare tumor markers among the three groups, the Kruskal-Wallis test was conducted, and logistic regression analysis was performed to adjust for multiple factors. *Post hoc* analyses were conducted using the Bonferroni correction to determine specific group differences, with p<0.05 set as the significance threshold to minimize Type I error due to multiple comparisons. All p-values were assessed using a two-sided approach.

## RESULTS

A total of 750 adult male participants were included in this study, equally distributed among three groups: 250 CC smokers, 250 any HTP users, and 250 quitters. The mean age was similar across groups (aged 26–72 years). BMI (kg/m^2^) was slightly higher in quitters (25.7 ± 3.4) than in CC (25.0 ± 3.3) and HTP smokers (25.5 ± 3.2). Systolic blood pressure was highest in quitters (127.3 ± 13.0 mmHg), while diastolic blood pressure was highest in any HTP smokers (79.7 ± 10.5 mmHg). Any HTP smokers had the longest smoking duration of a median of 25 years, whereas CC smokers had used them for a duration of a median of 16 years. Quitters had a median quit duration of 3 years. For tumor markers, AFP was evaluated in 747 subjects, CA 19-9 in 740 subjects, CEA in 739 subjects, and PSA in 742 subjects. AFP levels were highest in the any HTP smokers’ group (median: 3.2), while CA 19-9 levels peaked in the quitters’ group (median: 7.85). Additionally, CEA levels were greatest in the CC smokers’ group (median: 2.4), and PSA levels were highest in the HTP smokers’ group (median: 0.86). No statistically significant differences were found between the groups in terms of age, height, weight, BMI and systolic or diastolic blood pressure (Table 1).

**Table 1:** Participant characteristics by smoking status exposure group, among Saudi males (N=750)

*Variables*	*CC smokers* *(N=250)*	*Any HTP smokers* *(N=250)*	*Quitters* *(N=250)*
	*Mean ± SD*	*Mean ± SD*	*Mean ± SD*
Age (years)	48.9 ± 9.0	48.8 ± 9.1	49.1 ± 10.0
Height (cm)	172.8 ± 6.1	172.3 ± 5.8	171.9 ± 5.9
Weight (kg)	75.0 ± 11.8	5.9 ± 11.6	76.2 ± 11.7
BMI (kg/m^2^)	25.0 ± 3.3	25.5 ± 3.2	25.7 ± 3.4
Systolic blood pressure (mmHg)	124.4 ± 15.2	125.9 ±12.3	127.3 ± 13.0
Diastolic blood pressure (mmHg)	78.5 ± 11.4	79.7 ± 10.5	79.2 ± 10.9
	***Median (IQR)***	***Median (IQR)***	***Median (IQR)***
Duration of CC or HTP smoking (years), median (IQR)	16 (1–23)	25 (15–30)	15.5 (10–20.75)
Duration of HTP smoking (years), median (IQR)	-	2 (1–3)	1.5 (1–3)
Number of CCs used per day	17.5 (15–20)	15 (10–20)	10 (10–10)
Number of HTPs used per day		10 (10–20)	10 (10–10)
Quit duration (years)	-	-	3 (1-5)
AFP	3.1 (2.3–4.3)	3.2 (2.3–4.6)	2.9 (2.0–4.2)
CA 19-9	7.05 (4.80–9.90)	7.35 (4.93–11.60)	7.85 (4.90–11.00)
CEA	2.4 (1.6–3.5)	2.0 (1.3–2.7)	1.6 (1.0–2.28)
PSA	0.80 (0.56–1.16)	0.86 (0.60–1.19)	0.74 (0.50–1.03)

CC: combustible cigarette. HTP: heated tobacco product. BMI: body mass index. AFP: alpha-fetoprotein. CA 19-9: carbohydrate antigen 19-9. CEA: carcinoembryonic antigen. PSA: prostate-specific antigen. IQR: interquartile range.

**Figure 1:**
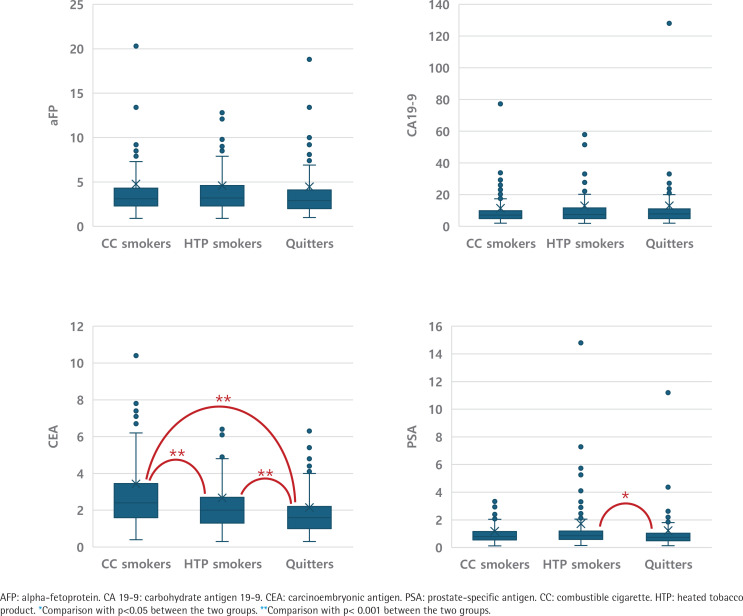
Kruskal-Wallis analysis of tumor markers according to type of cigarette

A Kruskal-Wallis analysis was conducted to evaluate differences in tumor marker levels among the cigarette type groups, including AFP, CA 19-9, CEA, and PSA. The results are presented in Figure 1. CEA was significantly higher in CC smokers compared to both any HTP users and quitters (p<0.001), with any HTP users also showing higher levels than quitters (p<0.001). PSA levels were significantly higher in any HTP users compared to quitters (p=0.002).

Logistic regression analysis showed that CC smokers had significantly higher odds of elevated CEA levels (OR=2.92; 95% CI: 1.95–4.36, p<0.001) compared to quitters, while HTP smokers also exhibited increased CEA levels (OR=1.71; 95% CI: 1.15–2.54, p<0.05). However, after adjustment, the significant difference in PSA levels between the any HTP smokers’ and quitters’ groups, observed in the Kruskal-Wallis analysis, was no longer apparent in the logistic regression analysis (Table 2).

**Table 2:** Logistic regression analysis of factors associated with tumor markers among Saudi males (N=750)

*Variables*	*AFP (N=747)*	*CA 19-9 (N=740)*	*CEA (N=739)*	*PSA (N=742)*
Cutoff value, median	7.0	34	5.5	3.0
	***OR (95% CI)***	***OR (95% CI)***	***OR (95% CI)***	***OR (95% CI)***
Age (years)	1.03 (1.00–1.05)*	1.04 (1.012–1.063)*	1.04 (1.01–1.06)*	1.01 (0.98–1.03)
BMI	0.97 (0.92–1.02)	0.97 (0.93–1.02)	1.00 (0.96–1.05)	0.96 (0.91–1.01)
SBP	1.00 (0.99–1.01)	1.01 (0.99–1.02)	1.00 (0.99–1.01)	1.00 (0.99–1.01)
Duration of smoking (years)	1.01 (0.98–1.03)	0.98 (0.99–1.01)	1.01 (0.99–1.037)	1.01 (0.98–1.03)
CC smokers	1.18 (0.81–1.74)	0.81 (0.56–1.20)	2.92 (1.95–4.36)**	1.20 (0.82–0.76)
HTP smokers	1.42 (0.97–2.08)	1.02 (0.69–1.50)	1.71 (1.15–2.54)*	1.38 (0.99–2.03)
Quitters ®	1	1	1	1

AFP: alpha-fetoprotein. CA 19-9: carbohydrate antigen 19-9. CEA: carcinoembryonic antigen. PSA: prostate-specific antigen. BMI: body mass index. SBP: systolic blood pressure. CC: combustible cigarette. HTP: heated tobacco product.

* p<0.05,

** p<0.001.

® Reference category.

## DISCUSSION

Evaluating the impact of HTP use on cancer-related biomarkers is crucial for understanding their potential role in public health interventions. Although HTPs are often considered a compromise for those unable or unwilling to quit nicotine entirely, their health effects must be critically examined. This study provides insights on the association of HTPs, CCs and quitting on cancer biomarkers. Such insights are essential for guiding public health policies and supporting individuals in making informed decisions regarding smoking and cessation strategies. This study aimed to determine how these different tobacco use patterns influence tumor markers, including AFP, CA 19-9, CEA, and PSA.

CEA and PSA levels were significantly different among the groups, with CC smokers exhibiting the highest CEA levels, followed by any HTP users and then quitters. In additional analyses, we observed a trend toward decreasing CEA levels with longer HTP use duration among HTP users, although this finding was not statistically significant. Elevated CEA is indicative of increased cancer risk, particularly for cancers such as lung, colorectal, and gastrointestinal cancers^2,14^. Our study demonstrated significant differences in biomarker levels among the groups. Although HTP smokers exhibited lower CEA levels than conventional cigarette smokers, the cross-sectional design and the heterogeneous characteristics of the HTP groups limit our ability to draw definitive conclusions regarding the assessment of any risk. Furthermore, while quitters displayed the lowest CEA levels –which may suggest a benefit of complete cessation – these findings should be interpreted with caution and warrant further investigation. PSA levels were also significantly higher in any HTP users compared to quitters. While PSA levels are not exclusive indicators of cancer, elevated PSA is associated with a higher risk of prostate abnormalities, including inflammation and cancer^16,17^.

These results may have important implications for public health policy. The elevated tumor marker levels observed in both CC smokers and HTP users suggest that both products are involved with unidentified factors related to CEA. The degree of this involvement appears to be greater in CC smokers, as indicated by their higher CEA levels. Complete smoking cessation is associated with the lowest CEA levels. While it is clear that smoking is closely linked to cancer, the direct impact of elevated tumor markers on cancer risk remains uncertain and warrants further investigation.

### Limitations

This study has several limitations that should be considered. First, the cross-sectional design limits the ability to establish causal relationships between tobacco use and tumor marker levels. Longitudinal studies are needed to determine whether the observed differences in tumor markers translate into differences in actual cancer incidence over time. Second, this study focused only on male participants, which limits the generalizability of the findings to females. Future research should include female participants to explore potential gender differences in the impact of tobacco use on tumor markers.

Furthermore, we were unable to investigate all potential factors that could influence each tumor marker. For example, PSA levels may vary based on prostate size or medications for benign prostatic hyperplasia. And various benign conditions can affect tumor marker levels. As a result, the inability to control these factors may have introduced bias.

Additionally, the study did not account for all potential confounding factors, such as diet, alcohol consumption, and physical activity, which could also influence tumor marker levels. Future research should consider these factors to provide a more comprehensive understanding of the risks associated with tobacco use. Moreover, the study did not distinguish between exclusive HTP users and those who use both HTPs and conventional cigarettes, which prevents us from isolating the effects of HTPs alone. Finally, the duration of tobacco use and the intensity of HTP use were self-reported, which may introduce recall bias. Objective measures of tobacco exposure could provide more accurate assessments in future studies.

Future research should also explore the biological mechanisms underlying the differential impacts of CC and HTP use on tumor markers. Understanding the specific pathways through which these products influence cancer risk could help in developing targeted interventions to reduce harm. In addition, studies investigating the impact of dual use (both CC and HTP) would provide valuable insights, as dual use is common among smokers transitioning to alternative products.

## CONCLUSIONS

This study shows that both conventional cigarette (CC) smoking and heated tobacco product (HTP) use were associated with elevated tumor marker levels, such as CEA and PSA, compared to individuals who have completely quit smoking. However, caution is warranted since the direct link between tobacco-induced increases in tumor markers and a higher cancer risk has not yet been established. As more studies comparing HTPs and CCs are conducted, our understanding of their effects is likely to improve, which may influence recommendations regarding health behaviors.

## Data Availability

The data supporting this research are available from the authors on reasonable request.
